# Hepatocellular Carcinoma (HCC) in Egypt: A comprehensive overview

**DOI:** 10.1186/s43046-020-0016-x

**Published:** 2020-01-16

**Authors:** Wafaa M. Rashed, Mohamed Ahmed Mohamed Kandeil, Mohamed O. Mahmoud, Sameera Ezzat

**Affiliations:** 1 Department of Research, Children’s Cancer Hospital-57357, Cairo, Egypt; 2 Department of Biochemistry, Faculty of Veterinary Medicine, Beni-Suef University, Beni-Suef, Egypt; 3 Department of Biochemistry, Faculty of Pharmacy, Beni-Suef University, Beni-Suef, Egypt; 4 Department of Epidemiology and Prevention Medicine, National Liver Institute, Menoufia University, Menoufia, Egypt

**Keywords:** Hepatocellular carcinoma, Epidemiology, Screening and Surveillance, Prevention, Diagnosis and treatment, Research

## Abstract

**Background:**

Worldwide, hepatocellular carcinoma (HCC) is a universal problem and its epidemiological data showed variation from place to place. Hepatocellular carcinoma (HCC) is the sixth and fourth common cancer in worldwide and Egypt, respectively. Egypt ranks the third and 15^th^ most populous country in Africa and worldwide, respectively. The aim of this review is to compare the status of HCC in Egypt to that in the worldwide from different issues; risk factors, screening and surveillance, diagnosis and treatment, prevention, as well as research strategy.

**Main body:**

The risk factors for HCC in Egypt are of great importance to be reported. The risk factor for HCC are either environmental- or host/genetic-related risk factors. In the last years, there is a tangible improvement of both screening and surveillance strategies of HCC in Egypt. The unprecedented national screening campaign launched by the end of 2018 is a mirror image of this improvement. While the improvement of the HCC prevention requires the governmental health administration to implement health policies. Although the diagnosis of Egyptian HCC patients follows the international guidelines but HCC treatment options are limited in terms of cost. In addition, there are limited Egyptian reports about HCC survival and relapse. Both basic and clinical HCC research in Egypt are still limited compared to worldwide.

**Short conclusion:**

Deep analysis and understanding of factors affecting HCC burden variation worldwide help in customization of efforts exerted to face HCC in different countries especially large country like Egypt. Overall, the presence of a research strategy to fight HCC in Egyptian patients will help in the optimum allocation of available resources to reduce the numbers of HCC cases and deaths and to improve the quality of life.

## Background

Worldwide, hepatocellular carcinoma (HCC) is a universal problem and its epidemiological data showed variation from place to place. Egypt ranks the third and 15^th^ most populous country in Africa and worldwide, respectively. In Egypt, the health authorities consider HCC as the most challenging health problem. The number of HCC patients increased twofold over a decade [1]. The aim of this review is to compare the status of HCC in Egypt to that in the worldwide from different issues; risk factors, screening and surveillance, diagnosis and treatment, prevention, as well as research strategy. Deep understanding of these issues help in customization of efforts exerted to face HCC in different countries especially large country like Egypt.

## Epidemiology and disease burden in Egypt

Hepatocellular carcinoma (HCC) represents the sixth most common cancer worldwide [2]. In Egypt, it represents the fourth common cancer [3]. Many hospital-based studies [1, 4–6] reported increasing the incidence of HCC. The reason for increased incidence could be attributed to (1) improvement in screening programs and diagnostic tools [7], (2) increasing the survival rate of cirrhotic patients that increases the chance of developing HCC, and (3) increasing the incidence and complications of hepatitis C virus (HCV) [4] which is the most important risk factor in developing liver cancer including HCC in Egypt [8].

Worldwide, HCC is the fourth most common cause of death from cancer [9]. It was estimated to be responsible for nearly 9.1% of the total deaths in 2012 (746,000 deaths) [10]. In Egypt, It is the most common cause of mortality- and morbidity-related cancer.

### Risk factors/etiology

There are many risk factors that play an important role in the development of HCC. These risk factors are summarized in Table 1.

**Table 1:** Risk factors for HCC development

I-Environmental-related risk factors		II-Host/genetic-related risk factors	
1- Infectious	a)- HBV b)- HCV	1- Host-related risk factors	a)- Gender b)- Ethnicity c)- Obesity d)- Diet e)- Oral contraceptives (OCs) f)- Autoimmune Hepatitis (Cryptogenic cirrhosis) g)- Diabetes Mellitus h)- NAFLD
2- Non-infectious	a) Chemical compounds b) Alcohol abuse c) Smoking tobacco	2- Genetic-related risk factors	i)- Monogenic risk factors: a)- Alpha 1 antitrypsin deficiency b)- Hemochromatosis
			ii)- polygenic risk factors: a)- Family history b)- Aflatoxins

### (I) Environmental-related risk factors

#### 1. Infectious risk factors

Both hepatitis B virus (HBV) and hepatitis C virus (HCV) increase HCC risk by 20-fold [11].

- HBV infection

Worldwide, HBV is one of the infectious risk factors of HCC. It accounts for 88% of cirrhosis-related HCC [12]. There are two patterns of HBV transmission: vertical transmission (from mother to newborns) and horizontal transmission (sexual and parenteral routes) [13]. HBV is oncogenic virus that integrates its genome in the host genome leading to both downregulation of tumor suppressor genes and activation of oncogenes [14]. In Egypt, there has been a decline in the prevalence of HBV infection over the last 20 years due to successful nationwide vaccination strategy [15, 16].

(b) HCV infection

Globally, HCV infection is the leading cause of cirrhosis (93%) [12] which is a risk factor for HCC [17]. It induces both hepatic inflammation and fibrosis. Mutation and malignant transformation of the infected cells are promoted by the HCV protein expression [14, 18, 19]. HCV infection is characterized by its long time progression to cirrhosis-related HCC [20]. Based on the phylogenetic and sequence analyses of HCV genomes, there are seven genotypes of HCV strains and 67 subtypes upon further classification of each genotype [21]. HCV genotype 4 is considered the most predominant HCV genotype in Egypt [22]. In Egypt, HCV prevalence may be attributed to the initiation of the mass schistosomiasis treatment campaigns in the 1950s and the 1960s [23]. Different HCV prevalence in Egypt were reported. The HCV prevalence in the age group (15–59 years) was 14.7% in 2008 while it became 10% in 2015. This decline in prevalence was related to aging of infected people receiving anti-schistosomal injections [24, 25].

#### 2. Non-infectious risk factors

Many environmental risk factors which is non-infectious play role in HCC risk.

- Chemical compounds

Occupational activities may include work exposure to a variety of chemical compounds. Liver is an important organ involved in detoxification, metabolic and excretory processes [26]. Therefore, HCC can be caused by the adverse effects of organic and inorganic chemical compounds exposure of the liver.

Inorganic compounds that may act as a risk factor for developing HCC include Arsenic [27] and Cadmium [28]. There are also number of *organic compound s*[26, 28] that may increase the risk of HCC. The most common are vinyl chloride monomer (VCM) and polyvinyl chloride (PVC) in addition to organic solvents (OS) that include trichloroethylene (TCE), perchlorethylene (PCE), N-nitrosamines, dioxin-like compounds (DLC), polychlorinated biphenyls (PCB), and polybrominated biphenyls (PBB). In addition to chloral and chloral hydrates that were predominately used in DDT and other insecticides. The ortho-toluidine (O-toluidine) is used in herbicides and pesticides. In Egypt, about (26%) of the population work in agriculture. This sector is at high risk for pesticides exposure and consequently high risk for developing HCC especially among rural males in addition to exposure to other risk factors (HBV and HCV) [4, 6, 29, 30]. In mid delta region in Egypt, both pesticides and fertilizers have been suggested to be an independent risk factor for HCC [31].

(b) Alcohol

Chronic alcohol intake is one of the known risk factor for HCC in many countries but it is extremely low in Egypt [6, 29, 30, 32]. The HCC risk increases by nearly five fold upon alcohol consumption of > 80 g/day ethanol for at least 5 years [33]. The underlying mechanism of developing HCC is complex and multi-factorial process [34].

(c) Smoking

In general, the tobacco ingredients are metabolized in liver and their carcinogenic effect is well-documented. Recently, a systematic review of 81 epidemiological studies [35] showed that there is an increase in the incidence of HCC risk and mortality among cigarette smokers. In Egypt, conflicting results were found regarding the association between tobacco smoking and the overall risk of HCC [5, 30, 31].

### (II) Host-/genetic-related risk factors

#### 1. Host-related risk factors

- Gender

There is gender variation of HCC incidence being the fifth most common cancer in men (7.5%) and the ninth in women (3.4%) [10]. In Egypt, HCC ranks the second and the sixth cancer in men and women, respectively [30]. This gender variation can be explained based on two reasons: biological reasons and environmental reasons. Biological reason for the variation of HCC incidence in women is explained by the level of estrogen hormone. It partially plays a role in suppression of interleukin (IL)-6-mediated inflammation that reduces both compensatory proliferation and liver injury [36]. Whereas testosterone in men can increase signaling of androgen receptors leading to promoting liver cell proliferation [37, 38]. This is in addition to variation in epigenetics and immune response. The environmental HCC incidence variation is explained by higher rate of men exposure to liver carcinogens such as occupational exposure to chemical compounds, alcohol and smoking in addition to hepatitis viral infection than women [4, 39]

(b) Ethnicity

Worldwide, there is racial disparity of HCC rate among population living in the same region [13]. In general, the heterogeneous distribution of HCC at regional and international level can be explained on the basis of differences in both the prevalence and the acquisition period of key risk factors for liver diseases in general and HCC in particular [1, 8, 32, 40].

(c) Obesity

It is a metabolic defect defined as body mass index (BMI) ≥ 30 kg/m^2^ and it is accompanied with an increase in the HCC risk by 89% [41, 42]. In Egypt, according WHO statistics 2008, there were approximately 46.3% females who are obese in comparison to 22.5% of males [30].

(d) Diet

Although a case-control study in Greece did not show any effect of diet (specific food category or certain nutrients) on the etiology of HCC [43], other studies showed this effect. An Italian case-control study [44] showed that there was an inverse relation between HCC risk and diet rich in both linoleic acid and β-carotene. Also, another Italian case-control study [45] showed the favorable effect of high intake of specific food for individuals at high risk for HCC. A Japanese study [46] among atomic bomb survivors showed 50% reduction in HCC risk in those subjects whose diet was high in isoflavone-rich miso soup and tofu. Research studies that investigate the association between diet and HCC risk in Egypt are lacking.

(e) Oral contraceptives

Although, there were two meta-analysis studies [47, 48] that investigated the association between HCC risk and oral contraceptives (OCs) use, the report showed inconclusive result about the association.

(f) Autoimmune hepatitis

Autoimmune hepatitis (AIH) is an immune-mediated inflammatory disease in liver. The association of AIH with limited polymorphisms at human leukocyte antigen (HLA) locus on chromosome 6p21.3 was confirmed. AIH progresses to cirrhosis which is the sine qua non for HCC with rate 1.1% in both sexes [42]. The curative treatment for both AIH and the underlying HCC is liver transplantation [49]. In Egypt, the incidence of HCC due to AIH is not available yet.

(g) Diabetes

Many genome-wide association studies (GWAS) have identified many loci that affect the risk of type 2 diabetes [50, 51]. There are many hypotheses [52] that explain the association between diabetes and the increased risk of HCC. Diabetes is one of the component of metabolic syndrome that may lead to non-alcoholic steatohepatitis (NASH) and consequently HCC. Also, persistent increase in insulin level in type 2 diabetic patients leads to both insulin resistance (IR) and an increase in the level of insulin-like growth factor-1 (IGF-1) in most tissues including liver that may accelerate carcinogenesis. In addition, chronic hyperglycemia may cause both oxidative stress and damage of hepatocytes. Also, a molecular mechanism involved in this association was observed upon detection of a p53 mutation (an apoptotic factor) in HCC diabetic patients compared to non-diabetics [53]. The prevalence of diabetes among HCC Egyptian patients has been reported by many studies [4, 5, 30, 54, 55] and a study confirmed association of type 2 diabetes increases the risk of HCC by 2–3-fold [5]. In type 1 diabetes, its association with HCC risk is still controversial [56–58].

(h) Nonalcoholic fatty liver disease

It is characterized by abnormal increase of hepatic triglycerides (> 5%) without extra alcohol intake [59]. In general, nonalcoholic fatty liver disease (NAFLD) increases the risk of HCC through developing NASH. In NASH patients, HCC is independent risk factor for mortality with hazard ratio = 7.9 [60]. Many genetic polymorphisms have been reported to be associated with NASH. In patients who have not consumed alcohol, NAFLD spectrum range from fatty liver to NASH that may end with cirrhosis. Worldwide, there are 20% of adults diagnosed with NAFLD, whereas up to 3% of adults are diagnosed with NASH [61]. In Egypt, an epidemiological study was conducted over 15 years on HCC patients and revealed that 5.3% of patients suffered from NASH [30]. This percentage is higher than the worldwide report.

#### 2. Genetic-related risk factors

They are classified into monogenic and polygenic risk factors.

(i) Monogenic risk factors

i. α1-Antitrypsin deficiency

α1-Antitrypsin deficiency (A1ATD) is hereditary metabolic syndrome. It is an autosomal recessive disease that originate from several mutations in the SERPINA1 gene located on chromosome 14q32.1. It is characterized by abnormal accumulation of A1AT protein/ SERPINA1 in endoplasmic reticulum in the liver that damages hepatocytes causing cirrhosis and finally HCC [49, 62]. It is associated with an increase in HCC risk especially in men [odds ratio (OR) = 5.0] [63]. However, the exact prevalence of A1ATD among Egyptian patients has not been estimated.

ii. Hereditary hemochromatosis or dietary iron overload

It can lead to excessive accumulation of iron in liver and consequently affect adversely hepatocytes such as (chronic necroinflammatory hepatitis then fibrosis and in some cases causing cirrhosis). Hemochromatosis protein (HFE) in 90% of hemochromatosis individuals is homozygous mutation at position 282 with substitution of tyrosine for cysteine (C282Y). HFE gene is positioned on chromosome 6p21.3 and is inherited as an autosomal recessive trait. Recent data suggests a 20-fold increased risk of HCC among hereditary hemochromatosis patients [64]. In Egypt, the estimated prevalence of hereditary hemochromatosis is 0.5% [30].

ii. Polygenic risk factors

- Family history of HCC

The association of family history of HCC to the HCC risk has been reported through heritable factors and modified by environmental factors [65]. In Egypt, 21.4% of HCC patients have a family history (first and second degrees relatives) of HCC [5].

(b) Aflatoxins

The global burden of aflatoxin-induced HCC ranges between 4.6 and 28.2% [66]. There are many studies conducted in Egypt that confirmed the presence of both aflatoxin-albumin adducts in human blood [67, 68] and aflatoxin in food [69]. Aflatoxins are carcinogenic metabolites of certain fungi called *Aspergillus flavus* and *Aspergillus parasiticum* that contaminate many agricultural crops especially maize, peanuts, and cottonseed. The aflatoxins from these crops play an important role in the incidence of hepatocarcinogenesis worldwide [26, 28] and also in Egypt [70]. The World health organization (WHO) classified aflatoxins as group 1 carcinogen [71]. The most carcinogenic type of aflatoxin is Aflatoxin B1 (AFB1). The genetic hallmark of AFB1 exposure and HCC risk is a specific mutation as a single-base substitution at the third base of codon 249 in the *TP53* gene. This mutation replaces an arginine by a serine (*R249S*) [72–74]. In addition, genetic polymorphism in the enzymes of activation (CYP enzymes) and deactivation (glutathione *S*-transferase) of pro-mutagenic aflatoxins may affect the level of pro-mutagenic aflatoxins and consequently the HCC risk [65].

It should be noted that immigration to Egypt after revolutions and wars in the Middle Eastern countries in the recent years may have an impact on all these risk factors. Screening for immigrants regarding the HCC risk factors, in general, and both HBV and HCV, in particular, should be encouraged.

## Screening and surveillance of HCC

There are conflicting reports about the impact of HCC detection at an early stage on both the cure rate and the overall survival (OS) [11, 75]. Globally, HCC surveillance include both ultrasound and alpha fetoprotein (AFP) level measurement [76].

Several guidelines are available for screening high-risk populations including those diagnosed with cirrhosis and/or HBV/HCV infection (with or without cirrhosis) [11]. Screening methods and surveillance intervals are the main differences between these guidelines. Although these guidelines affected greatly medical practice but due to poor adherence to screening, HCC mortality worldwide is increasing [11].

In Egypt, a national screening campaign was started by the Egyptian Ministry of Health (MOH) in 2018 to combat high HCV prevalence in Egypt by 2020 [77]. All screened participants with confirmed HCV infection are enrolled in government-subsidized treatment program using direct acting antiviral (DAA); sofosbuvir-based regimen. However, a nationwide campaign for HCC surveillance is still not available. Many studies showed conflicting results regarding the outcome of DAA treatment and HCC recurrence exit. Given the size of the HCV and HCC problems in Egypt, the HCV treatment program could yield important results on the efficiency of HCV treatment using DAA agents on HCC risk in the near future [78–81].

## Diagnosis and treatment approach

### Diagnosis

During surveillance, finding a suspicious lesion using ultrasound in cirrhotic liver is followed by diagnostic confirmation using contrast enhanced helical computed tomography (CT) or dynamic magnetic resonance irradiation (MRI). Also, non-pathological confirmation of HCC diagnosis is achieved by AFP testing combined with previously mentioned imaging techniques [82].

#### HCC treatment centres in Egypt

There are seven types of centers that diagnose and treat HCC patients in Egypt:

1. Liver institutes that affiliated to Egyptian Ministry of Health (MOH), Universities, and Non-governmental organizations (NGO).
2. Cancer centers affiliated to Egyptian MOH: there are ten specialized oncology center till now in nine governorates.
3. Cancer centers affiliated to Ministry of Higher Education.
4. Oncology/hepatology/tropical units in MOH and university hospitals.
5. NGO Cancer Centers: there is only one in the upper Egypt.
6. Military oncology Units that treat both military and civilian patients.
7. Private cancer centers and oncology clinics inside private hospitals.

The geographical distribution of these treatment centers all over Egypt should be assessed in relation to healthcare service provision, and the heterogeneity of patients’ flow from different governorates. This will help in balanced geographical distribution of healthcare system.

### Treatment approach

**Fig. 1:**
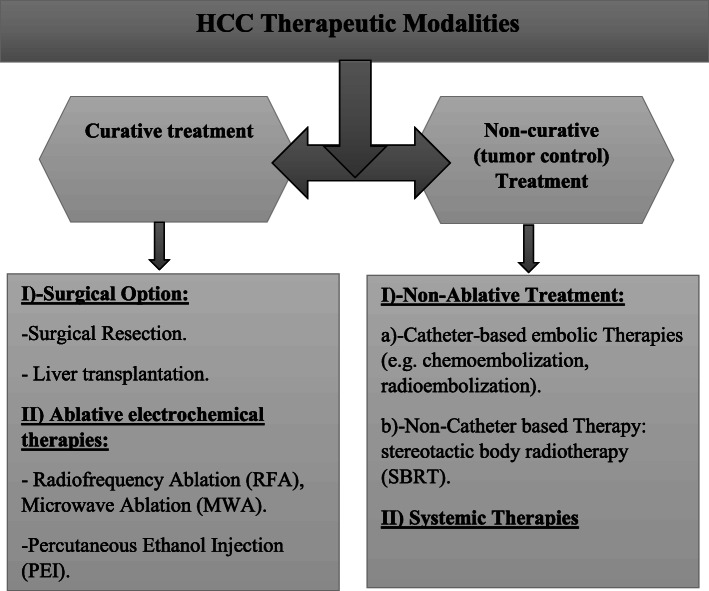
Therapeutic modalities used for HCC treatment

Precise staging of HCC initially is very useful for determination of the therapeutic options and the overall prognosis of the disease. There are certain clinical features upon which most staging systems use for HCC assessment. These clinical features are size and local extent of the tumor, metastasis of the tumor, severity of the liver disease, and the overall patient performance status [83]. There are two common staging systems; (a) American Joint Committee on Cancer (AJCC) Tumor-Node-Metastasis (TNM) [84] and (b) The Barcelona Clinic Liver Cancer (BCLC) system [84]. The first system characterizes both tumor features, lymph node involvement, and metastases. While the second system depends on the combination of tumor features, severity of the liver disease, and patient performance status. In comparison to other prognostic systems, BCLC system has the best correlation with the patient outcome [85]. Figure 1 represents different options for HCC therapeutic modalities used. Recently, results of studies for HCC treatment and prevention of complication have been reported [86–89]. Details about clinical trials in Egypt will be discussed in HCC research section.

The median overall survival (OS) of late diagnosis of HCC ranges between 6 and 20 months. In US, the 2- and 5-year OS were < 50% and 10%, respectively [90]. In Egypt, HCC survival and relapse are not reported extensively. The median OS of untreated HCC patients was 2.3 months [91]. Although all reports about the OS for treated HCC patients were slightly different but the OS is still poor [92, 93].

Many systemic cytotoxic chemotherapy drugs are used in HCC treatment as single agents, e.g.,: cisplatin, doxorubicin, 5-flurouracil, or combined regimen. All these chemotherapeutic agents are available in Egyptian market. These systemic treatments had three main disadvantages [94, 95]: (1) They have between 10 to 25% response rate with marginal survival improvement, (2) patients with underlying liver cirrhosis are poorly tolerating these treatments, and (3) HCC is highly resistance to single agent regimen.

Currently, there is paradigm shift in the HCC treatment by the introduction of immune checkpoint inhibitors in addition to molecular targeted therapies [96]. Many therapeutic agents for HCC are targets to different pathways implicated in hepatocarcinogenesis. Table 2 represents the list of FDA-approved targeted and immune therapies for HCC that are available in Egyptian market. In fact, there is an economical burden in the treatment of HCC in general and in using these expensive targeted therapies in particular. So, using targeted therapies in HCC treatment for Egyptian patients are limited to patients who can afford the cost.

**Table 2:** FDA-approved targeted and immune therapies for HCC

Generic drug	Brand name	Target	Availability in Egyptian Market
Cabozantinib	Cabometyx and Cometriq	Multikinase inhibitor	Available
Lenvatinib	Lenvima	Multikinase inhibitor	Available
Sorafenib	Nexavar	Multikinase inhibitor	Available
Regorafenib	Stivarga, and Regonix	Multikinase inhibitor	Available
Nivolumab	Opdivo	Immune check point inhibitors	Available
Pembrolizumab	Keytruda	Immune check point inhibitors	Available
Ramucirumab	Cyramza	Human monoclonal antibody against vascular endothelial growth factor 2(VEGFR 2)	Available

## Prevention of HCC

Clear determination of HCC risk factors is very helpful for well-designed strategies for HCC prevention. Generally, prevention of HCC is based on early prevention of HCC risk factors (primary prevention), treatment of risk factors at an early stage (secondary prevention), and preventing or decreasing HCC relapse after successful curative treatment (tertiary prevention) [97, 98].

There are different methods for HCC primary prevention. Routine HBV immunization to all newborns (within 24 h) and high-risk groups is recommended by WHO [99]. This universal vaccination along with other behavioral pattern changes that decrease the risk of infection are very important primary prevention together with implementation of surveillance programs. In addition, antiviral treatment for chronic HBV and HCV patients is used for HCC secondary prevention [97, 98].

In Egypt, there are two methodologies for HCC primary and secondary prevention; HBV vaccination program [16], and, recently, HCV eradication through national campaign [77]. On the other hand, the principle of HCC prevention through education is the number one recommendation by World Gastroenterology organization’s global guidelines [100]. Education intervention study as a pilot study had been conducted and showed promising results [101]. Designing an education-based intervention programs that show relation between the best preventive practice (e.g., pesticide handling and food storage) and HCC risk is highly needed. This is recommended especially for habitants of rural areas (high risk) [1].

In addition, HCC prevention should be supported by health care provider, patients, and health care system as a whole [98]. Each one has a definite responsibility. Health care provider who has a good knowledge should identify HCC risk factors and patients at risk then refer them for screening and surveillance. There is an Egyptian study that confirmed this role for academic physicians working in University Hospitals [102]. Patients should show compliance with health care provider recommendations. Furthermore, the health care system should have the capacity and responsibility to deliver surveillance tests. To sum up, it is the responsibility of governmental health administrations to implement health policies regarding HCC prevention.

## HCC research

Hepatocellular carcinogenesis has been attributed to many biological aberrations, e.g., mutations, epigenetic dysregulations, and chromosomal anomalies. Six predominant molecular pathways have been identified in HCC by whole-exome sequencing (WES). They include TERT promoter mutation, Wnt/β-catenin, the P53 cell-cycle pathway, epigenetic modifiers in histone methylation and chromatin remodeling, mutations in oxidative stress pathways (including NFE2L2 and KEAP1), PI3K/AKT/mTOR, and RAS/MAPK pathways [103]. This is in addition to various molecular pathways that were recently discovered in a large study conducted on 363 HCC cases using WES and DNA copy number analysis and on 196 HCC cases using DNA methylation, RNA, miRNA, and proteomic expression [104]. Recently, Calderaro and colleagues proposed molecular and clinical features-based classification for HCC [105]. A recent systematic review identified 544 articles (16.2%) published in PubMed about HCC in Egypt [106]. In Egypt, some abnormalities in molecular pathways involved in HCC have been identified [107, 108] but other abnormalities need to be identified on a large scale of HCC Egyptian patients using advanced technology. Identification of molecular characteristics of HCC Egyptian patients will pave the way for personalized therapy toward improvement of their overall survival [107].

### Clinical trials of HCC

Currently, in the era of precisian medicine, genomic profiling-based clinical trial has been started. NCI-MATCH (ClinicalTrials.gov Identifier: NCT02465060) is the largest precision medicine that started in July 2015 [109]. It contains different targeted-therapies for each genetic abnormality arm/group in its design. It enrolls patients with specific “matching” genetic aberration and irrespective of their cancer type. Recently, ComboMATCH is another example of precisian trial that will be conducted but for combined targeted agents.

In Egypt, there are ten interventional clinical trials registered on clinicaltrials.gov (Table 3). Out of the ten clinical trials, there are four clinical trials that used targeted therapies; two of them were terminated. One of them (NCT01539018) showed no evidence of difference between treatments used. The other one (NCT01009593) was terminated by a recommendation from Independent Data Monitoring Committee (IDMC). The concept of clinical trials in Egypt is still limited that is why there is a low number of clinical trials in HCC.

**Table 3:** HCC clinical Trials in Egypt

No.	Clinical trial number	Drug	Phase	Status
1	NCT02715492	LMWH	Phase 3	Not yet recruiting
2	NCT02771405	•Drug: Sofosbuvir •Drug: Ribavirin •Drug: Simeprevir •Drug: daclatasvir •Drug: Ledipasvir	Phase 3	Recruiting
3	NCT03551444	Administration of DAA-based treatment	Phase 3	Recruiting
4	NCT02971696	•Drug: Sorafenib •Drug: Best Supportive care	Phase 3	Completed
5	NCT03151213	•Drug: Pregabalin 150 mg •Other: Placebo	Phase 3	Recruiting
6	NCT02646137	•Drug: Transarterial chemoembolization (TACE) •Procedure: Radiofrequency ablation combined with TACE •Procedure: Microwave ablation combined with TACE	Phase 3	Recruiting
7	NCT01539018	•Drug: Sorafenib •Drug: sorafenib plus tegafur-uracil	Phase 2	Terminated
8	NCT02568748	•Biological: CIK •Procedure: TACE	Phase 3	Recruiting
9	NCT01655693	•Drug: Doxorubicin •Drug: Best Standard of Care	Phase 3	Active, not recruiting
10	NCT01009593	•Drug: ABT-869/Linifanib •Drug: Sorafenib	Phase 3	Terminated

In general, there is no structured national research program for HCC in place. The same notice was reported on liver research in Egypt [106]. There are many important research topics in HCC are still untouched deeply. The economic burden of HCC treatment and its relation to the health outcome, the effect of immigrants on HCC distribution in Egypt, the effect of diet on HCC risk, education-based intervention studies especially in rural area inhabitants (high risk), and molecular and epigenetic characteristics of HCC in Egyptian patients are good examples for these research topics.

Research in HCC can be stimulated at many levels. On the institutional level, forming coordinated multidisciplinary research team who will study different aspects of HCC (epidemiological, diagnostic, treatment, and palliation aspects). On national level, establishing Egyptian Research Network for HCC (EARN HCC) is highly recommended. It will ease linking between different HCC specialized institutions to foster application of their respective expertise accumulated over years. On the international level, collaboration between Egyptian institutions and peer international specialized HCC institutions in different domains (training, twining research) should be supported by government health administration.

## Conclusion

Hepatocellular carcinoma (HCC) is a universal problem and its epidemiological data showed variation from place to place. Deep analysis and understanding of factors affecting HCC burden variation worldwide help in customization of efforts exerted to face HCC in different countries especially large country like Egypt.

## Data Availability

Not applicable.

## Abbreviations

A1ATD: α1-antitrypsin deficiency
AFB1: Aflatoxin B1
AFP: Alpha fetoprotein
AIH: Autoimmune hepatitis
AJCC: American Joint Committee on Cancer
BCLC: The Barcelona Clinic Liver Cancer system
BMI: Body mass index
CT: Computed tomography
DAA: Direct acting antiviral
DLC: Dioxin-like compounds
EARN HCC: Egyptian Research Network for HCC
GWAS: Genome-wide association studies
HBV: Hepatitis B virus
HCC: Hepatocellular carcinoma
HCV: Hepatitis C virus
HFE: Hemochromatosis protein
HLA: Human leukocyte antigen
IDMC: Independent Data Monitoring Committee
IGF-1: Insulin-like growth factor-1
IL: Interleukin
IR: Insulin resistance
MOH: Ministry of Health
MRI: Magnetic resonance irradiation
NAFLD: Nonalcoholic fatty liver disease
NASH: Non-alcoholic steatohepatitis
NGO: Non-governmental organizations
OCs: Oral contraceptives
OS: Overall survival
O-toluidine: Ortho-Toluidine
PBB: Polybrominated biphenyls
PCB: Polychlorinated biphenyls
PCE: Perchlorethylene
PVC: Polyvinyl chloride
TCE: Trichloroethylene
TNM: Tumor-node-metastasis
VCM: Vinyl chloride monomer
WES: Whole-exome sequencing
WHO: World Health Organization
